# Future Guardians of Health: Involving The Young Generation in the One-Health Program in India

**DOI:** 10.3389/phrs.2026.1609453

**Published:** 2026-04-01

**Authors:** Kajal Srivastava, Shweta Chauhan, Sahjid Mukhida, Shaili Vyas

**Affiliations:** 1 Department of Community Medicine, Dr. D. Y. Patil Medical College Hospital and Research Centre, Dr. D. Y. Patil Vidyapeeth, Pune, Maharashtra, India; 2 Department of Microbiology, Dr. D. Y. Patil Medical College Hospital and Research Centre, Dr. D. Y. Patil Vidyapeeth, Pune, Maharashtra, India; 3 Department of Epidemiology and Public Health, Himalayan Institute of Medical Sciences, Dehradun, India

**Keywords:** children, one health, Public Health, school age children, young adolescents

Dear Editors

The growing threats of zoonotic diseases, antimicrobial resistance, and climate change have increasingly highlighted the interconnectedness of human, animal, and environmental health. Despite this global recognition, awareness of the One Health approach remains limited in early education systems. In this context, the recently published article titled “One Health Education for Children as a Catalyst for Systems Change and Climate Acton in Africa” presents a timely and important discussion on the role of educating children about One Health principles [1].

As described by Abia WA et al, the One Health concept emphasizes the interdependence between humans, animals, and the environment. Introducing these ideas during childhood has the potential to shape longe-term attitudes and behaviors towards health and sustainability. The authors effectively highlight how early education can serve as a catalyst for broader systems change and climate action. Their review also provides valuable insights into initiative undertaken by several countries to integrate one health concepts into educational frameworks [1].

However, in contrast to the growing global recognition of One Health, progress in implementing such initiatives in India has been relatively slow. One of the first efforts in this direction was the launch of a summer school on One Health in May 2025 aimed at empowering youth with knowledge of the One Health Approach [2]. Nevertheless, multiple challenges continue to hinder effective implementation. These include fragmentation across sectors that serve as key stakeholders in One Health, differences in institutional priorities, limited intersectoral data sharing, and legislative barriers that complicate coordinated action [3]. To address these gaps, Saxena et al have proposed an operationalization framework for implementing the One Health approach in India [4].

Several institutions in India have also initiated efforts to promote One Health research and capacity building. For instance, the Indian Institute of Public Health, Gandhinagar, in collaboration with the Institute of Hygiene and Public Health (IHPH), Bonn, Germany, established the center for One Health Education, Research and Development (COHERD). This center focuses on critical areas such as zoonotic disease control, pandemic preparedness, and antimicrobial resistance [5]. Additionally, previous publication have highlighted key stakeholders involved in the One Health ecosystem in India as well as national initiatives taken by the Government of India to promote this approach [6, 7].

India presents a unique contest for the implementation of One Health Principles. A large proportion of the population resides in rural areas where close interactions between humans, animals, and the environment are part of daily life. In many communities, animals are considered an integral part of the household, and children grow up in environments where human-animal interactions are common. This makes early education on One Health particularly relevant, as it can help shape responsible behaviors toward environment sustainability, animal health and human wellbeing.

Encouragingly, several awareness initiatives have recently been undertaken. Government agencies professional bodies such as the Indian Association of Preventive and Social Medicine (IAPSM) and institutions guided by the Indian Council of Medical Research (ICMR) observed November 2025 as “One Health Month.” During this period, a range of activities were organized to raise awareness about the One Health concept, including community awareness rallies, poster and infographic competitions, short awareness sessions, video campaigns, and continuing medical education programs for health professionals [8]. These initiatives play an important role in sensitizing both students and the broader community to the importance of integrated health approaches.

In conclusion, the One Health approach is increasingly recognized as essential for addressing current and future global health challenges. Integrating One Health education at an early stage of schooling could help nurture a generation that understands the interconnected nature of health systems and actively contributes to sustainable health practices. We therefore support the call for strengthening educational initiatives and policy efforts that promote One Health awareness among children and young people.

## References

[1] Abia WA Fomboh R Kognoudjui AM Abia EA . One Health Education for Children as a Catalyst for Systems Change and Climate Action in Africa. Public Health Rev (2025) 46:1608071. 10.3389/phrs.2025.1608071 41321977 PMC12660149

[2] SEAOHUN. News Release: India Launches First-Ever Summer One Health School Program. (2025). Available online at: https://www.seaohun.org/single-post/news-release-india-launches-summer-onehealth-school-program (Accessed November 21, 2025).

[3] Tiwari S Shewale A Singh A Nale T Mishra D . One Health in Action: National One Health Programme for Prevention and Control of Zoonoses - Need, Strategy, Progress, and Challenges 1 1 1 1 1 Available online at: https://ncdc.mohfw.gov.in/wp-content/uploads/2025/05/NCDC-Quarterly (Accessed November 21, 2025).

[4] Saxena D Yasobant S Kalpana P Sayed ZQ Bhardwaj P . Operationalization of One Health Approach in India: Still Miles to Go? Indian J Community Med (2024) 49(Suppl. 2):S202–S204. 10.4103/ijcm.ijcm_753_24 40124869 PMC11927819

[5] COHERD | Iiphg (2025). Available online at: https://iiphg.edu.in/coherd/ (Accessed November 21, 2025).

[6] Nambiar P . India to Envision One Health Movement for Confronting Emerging Health Threats: From Concept to Approach Toward Institutionalization. Int J One Health (2020) 6(2):165–76. 10.14202/ijoh.2020.165-176

[7] Yasobant S Memon F Pachillu K Saxena D . One Medicine Vs One Health: Policy Disconnect in India. CABI One Health (2024) 3:1. 10.1079/cabionehealth.2024.0001

[8] One Health IAPSM and ICMR Joint Initiative. (2025). Available online at: https://www.scribd.com/document/945530470/ONE-Health-Activities-Flyer (Accessed November 30, 2025).

